# EphA receptors and ephrin-A ligands are upregulated by monocytic differentiation/maturation and promote cell adhesion and protrusion formation in HL60 monocytes

**DOI:** 10.1186/s12860-017-0144-x

**Published:** 2017-08-29

**Authors:** Midori Mukai, Norihiko Suruga, Noritaka Saeki, Kazushige Ogawa

**Affiliations:** Laboratory of Veterinary Anatomy, Graduate School of Life and Environmental Sciences, Osaka Prefecture University, 1-58 Rinku-Ourai-Kita, Izumisano, Osaka, 598-8531 Japan

**Keywords:** Monocytes, HL60, EphA, Ephrin-A, Cell adhesion, Differentiation

## Abstract

**Background:**

Eph signaling is known to induce contrasting cell behaviors such as promoting and inhibiting cell adhesion/spreading by altering F-actin organization and influencing integrin activities. We have previously demonstrated that EphA2 stimulation by ephrin-A1 promotes cell adhesion through interaction with integrins and integrin ligands in two monocyte/macrophage cell lines. Although mature mononuclear leukocytes express several members of the EphA/ephrin-A subclass, their expression has not been examined in monocytes undergoing during differentiation and maturation.

**Results:**

Using RT-PCR, we have shown that EphA2, ephrin-A1, and ephrin-A2 expression was upregulated in murine bone marrow mononuclear cells during monocyte maturation. Moreover, EphA2 and EphA4 expression was induced, and ephrin-A4 expression was upregulated, in a human promyelocytic leukemia cell line, HL60, along with monocyte differentiation toward the classical CD14^++^CD16^−^ monocyte subset. Using RT-PCR and flow cytometry, we have also shown that expression levels of αL, αM, αX, and β2 integrin subunits were upregulated in HL60 cells along with monocyte differentiation while those of α4, α5, α6, and β1 subunits were unchanged. Using a cell attachment stripe assay, we have shown that stimulation by EphA as well as ephrin-A, likely promoted adhesion to an integrin ligand-coated surface in HL60 monocytes. Moreover, EphA and ephrin-A stimulation likely promoted the formation of protrusions in HL60 monocytes.

**Conclusions:**

Notably, this study is the first analysis of EphA/ephrin-A expression during monocytic differentiation/maturation and of ephrin-A stimulation affecting monocyte adhesion to an integrin ligand-coated surface. Thus, we propose that monocyte adhesion via integrin activation and the formation of protrusions is likely promoted by stimulation of EphA as well as of ephrin-A.

**Electronic supplementary material:**

The online version of this article (doi:10.1186/s12860-017-0144-x) contains supplementary material, which is available to authorized users.

## Background

Eph receptors and ephrin ligands are membrane proteins that primarily regulate cell-cell repulsion and adhesion as well as cell adhesion and movement by modulating the organization of the actin cytoskeleton mainly via Rho family GTPases [1]. In mammals, the Eph receptor tyrosine kinase family has 14 members that are divided into the EphA (A1–A8 and A10) and EphB (B1–B4 and B6) subclasses based on the sequence similarity of their extracellular domains. The members of these two receptor subclasses promiscuously bind the ligands of the ephrin-A (A1–A5) and -B (B1–B3) classes, respectively. Ephrin-A ligands are anchored to the plasma membrane through a glycosyl phosphatidylinositol linkage, whereas ephrin-B ligands are a class of transmembrane proteins. Interaction of Eph receptors with ephrin results in bidirectional signaling in both the receptor- and ligand-expressing cells. Forward signaling by Eph mainly depends on autophosphorylation and phosphorylation by other tyrosine kinases as well as by the association of the receptor with various signaling proteins, whereas reverse signaling by ephrin largely depends on Src family kinases [2, 3].

Integrins, a large family of cell adhesion molecules, bind to proteins as ligands in the extracellular matrix and on the cell surface. Integrins are heterodimeric transmembrane proteins composed of α and β integrin subunits, and 18 α subunits and 8 β subunits have been identified in humans to date, thus generating 24 heterodimers [4]. Notably, integrins can transform their conformation from a bent inactive form to an extended closed form (intermediate ligand affinity) and further to an extended open conformation (high ligand affinity) in response to stimulation from other receptors. Integrins play important roles during leukocyte chemotaxis, infiltration, and migration, and conformational changes have been studied intensively for the leukocyte integrin, LFA-1 (αLβ2). In this context, chemokine receptors, upon binding chemokines, rapidly induce integrin activation by activating Rap1 small GTPase, a key regulator of integrins and many other molecules involved in chemokine-driven signaling cascades in leukocytes [5–7]. These signals resulting in integrin activation are termed “inside-out signaling.” Once activated and bound to their ligands, integrins generate intracellular signals referred to as “outside-in signaling,” which in turn alter various cellular functions such as cell motility and proliferation involving the activation and/or recruitment of signaling molecules such as Src, phosphatidylinositol 3-kinase (PI3K), and Rho family GTPases. Moreover, in their activated state, integrins can regulate their own molecular configuration, such as their clustering and stabilization in focal adhesions, allowing them to continue the regulation of downstream signaling [5–7].

Evidence indicating crosstalk between Eph/ephrin signaling and integrin signaling has accumulated recently [3, 8]. Integrin inside-out and outside-in signaling involves the activation and/or recruitment of upstream/downstream signaling molecules such as Rap1, Rho family GTPases, Src, PI3K, and focal adhesion kinase (FAK), all of which also serve as key players in the downstream cascade mediated by Eph/ephrin signaling [3]. While these receptor-regulated pathways appear to overlap in some ways, to our knowledge, studies investigating the interaction between Eph/ephrin and integrin/integrin ligand signaling on cell adhesion are insufficient, particularly in terms of leukocytes and related cells, and many of the reported investigations are conflicting. For example, in human T cells, β1- and β2 integrin-mediated adhesion to integrin ligands has been shown to be stimulated by ephrin-A activation and inhibited by EphA [9]. However, ephrin-A signaling significantly reduced adhesion to integrin ligand-coated surfaces in addition to impairing chemokine-mediated trans-endothelial migration in chronic lymphocytic leukemia cells [10], whereas EphA signaling increased β1-integrin-mediated adhesion to an integrin ligand-coated surface in dendritic cells [11]. Thus, additional research is warranted to elucidate whether EphA and ephrin-A promote or inhibit integrin mediated cell adhesion specifically in mononuclear leukocytes (T cells, B cells, and monocytes) since these are known to express several members of the EphA/ephrin-A subclass [12–16]. The goal of this study is to clear up these uncertainties using a monocyte differentiation model cell line.

To our knowledge, EphA and ephrin-A subclass expression has not been examined in monocytes during differentiation even though mature monocytes express several members in the EphA/ephrin-A subclass [12]. The human promyelocytic leukemia cell line, HL60, has been used as a monocyte differentiation model for mechanistic studies, and monocytes differentiated from HL60 (HL60 monocytes) are frequently used as monocyte substitutes [17–19]. Thus, we examined the expression of EphA/ephrin-A in HL60 cells during monocytic differentiation as well as in bone marrow mononuclear cells during monocyte maturation. Recently, using a cell attachment stripe assay, we demonstrated that EphA2, upon stimulation with ephrin-A1, promotes cell adhesion through interactions between integrins and integrin ligands in monocyte/macrophage cell lines and, further, that the ectodomain of truncated EphA2 itself induces cell adhesion [20, 21]. Since monocytes express certain members of EphA as well as ephrin-A, we examined whether ephrin-A stimulation affects cell adhesion to integrin ligand-coated surface in HL60 monocytes. This is the first study demonstrating that certain members of EphA and ephrinA are upregulated during monocyte differentiation/maturation and that ephrin-A, upon stimulation with EphA2, likely promotes adhesion through integrin-integrin ligand interaction in monocytes.

## Methods

### Animals

Male ICR mice at 7 weeks of age, maintained under standard housing and feeding conditions, were used for isolation of bone marrow mononuclear cells (MNCs). The animal experimentation protocol was approved by the Animal Research Committee of the Osaka Prefecture University.

### Isolation of bone marrow mononuclear cells and their differentiation/maturation into adherent monocytes

The femurs, tibias, and humeri were aseptically removed from male ICR mice, and the epiphyses of the bones were dissected. Bone marrow cavities were flushed with ice-cold Hank’s balanced salt solution (HBSS; Sigma-Aldrich, St Louis, MO, USA) using a syringe and a 23-gauge needle to collect bone marrow cells (BMCs). MNCs were isolated by density-gradient centrifugation according to the method described by Graziani-Bowering et al. [22] with some modifications. In brief, BMCs were fractionated by equilibrium density centrifugation on a discontinuous gradient of an iodixanol solution (Opti-Prep; Axis-Shield, Oslo, Norway). A 4 mL mixture containing BMCs and an iodixanol solution at a density of 1.090 g/mL was prepared by dilution with RPMI-1640 medium (Sigma-Aldrich). An iodixanol solution of 4 mL diluted with the medium at a density of 1.080 g/mL was overlaid on the mixture in a 15 mL plastic centrifuge tube followed by addition of 0.5 mL HBSS. The tube was allowed to stand upright at 4 °C for several min and was then centrifuged at 100×*g* for 20 min at 4 °C. MNCs fractionated between the iodixanol solution and HBSS were then collected. To remove the adherent cells including mature monocytes and macrophages in this fraction, MNCs at a density of 1 × 10^6^ cells/mL were incubated overnight in a tissue culture dish with RPMI-1640 medium containing 10% heat-inactivated fetal bovine serum (FBS; Nichirei Biosciences, Tokyo, Japan), 100 U/mL penicillin, 100 μg/mL streptomycin (pen/strep; Sigma-Aldrich), and 5 ng/mL murine macrophage colony-stimulating factor (M-CSF; PeproTech, Rocky Hill, NJ, USA). Non-adherent MNCs were then seeded at a density of 3.2 × 10^5^ cells/mL, cultured in medium containing 20 ng/mL M-CSF, and allowed to propagate and differentiate into monocytes. At day 1 after seeding, adherent cells were collected as samples (MC-1d), and at day 2, non-adherent cells were discarded and adherent MNCs were cultured with fresh medium for 3 more days (MC-5d). Adherent MNCs detached from the dish surface by pipetting were collected by centrifugation and used for nonspecific esterase (NSE) staining to identify monocytes and for RT-PCR analyses for the expression of the monocyte differentiation marker CD115 [23, 24] and the undifferentiated myeloid cell marker CD34 [25] to estimate the differentiation states between groups, and among members of the EphA/ ephrin-A subclass.

### Differentiation of HL60 into monocytes

The human promyelocytic leukemia cell line, HL60, was obtained from the RIKEN BioResource Center (Ibaraki, Japan), cultured in suspension in RPMI-1640 supplemented with 10% FBS and pen/strep, and maintained in a 5% CO_2_ atmosphere at 37 °C.

HL60 cells have been widely used as terminal differentiation models of monocytes, with 1α, 25-dihydroxy-vitamin D_3_ (VD) and TNFα as inducers of monocytic differentiation. Therefore, HL60 cells were differentiated to monocytes by stimulation with VD and/or TNFα, in accordance with previous studies [17–19]. Cells were seeded at a concentration of 5 × 10^4^ cells/mL in a tissue culture dish, treated with 50 nM VD (Sigma-Aldrich) dissolved in ethanol, and cultured for 3 days to allow differentiation (VD group). In some dishes, TNFα at 5 ng/mL (Roche Diagnostics, Mannheim, Germany) was added 2 days after VD addition and culture continued for 1 day (VD-TNF group). Control cultures were treated with the same volume of ethanol, reaching less than 0.1% (*v*/v) of the final volume (control group). At 3 days after seeding, the non-adherent cells and the adherent cells detached from the dish by pipetting were collected by centrifugation. These cells were used for NSE staining to determine the frequency of monocyte differentiation as well as for RT-PCR analyses to compare the expression of monocyte markers among the three groups; CD14, CD16, and CD115 were used as monocyte markers [23, 24] and CD15 was used as a marker of undifferentiated hematopoietic cells [26]. Expression levels of EphA receptors and ephrin-A ligands as well as the various integrin α/ß chains were also determined by RT-PCR in HL60 cells from the control and VD-TNF groups. Moreover, cell surface expression of the integrin α/ß subunit proteins whose expressions were identified by RT-PCR was examined by flow cytometry.

### Nonspecific esterase staining analysis

Enzyme cytochemical staining for the fluoride-sensitive NSE enzyme, which has been widely used for identifying monocytes because of its simplicity, was performed to identify the adherent MNCs treated with M-CSF and the HL60 cells treated with VD and/or TNFα as monocytes according to the method described by Li et al. [27] with some modifications. Cell smears on glass slides were dried and fixed with 10% formalin and 60% acetone in PBS for 1 min at 4 °C. After washing with distilled water, the smears were incubated with a mixture of 10 mg/mL 1-naphthyl butyrate (Sigma-Aldrich) in ethylene glycol monomethyl ether (EGME; Sigma-Aldrich) and 0.5 mg/mL Fast Garnet GBC salt (Sigma-Aldrich) in 1/15 M phosphate buffer, pH 6.4 at a ratio of 1:20 for 40 min at 32 °C. Fluoride-sensitivity for NSE activity was verified by incubation in the reaction mixture with 40 mM sodium fluoride (Sigma-Aldrich) [28]. After washing, the cells were mounted in glycerol and photographed under a microscope (IX71; Olympus, Tokyo, Japan). To determine the differentiation efficiencies of HL60 cells treated with VD and/or TNFα, three random field images per smear sample were photographed for each group (control, VD, VD-TNF) using a 10 × objective lens. Cells stained brown to dark brown or almost black were defined as NSE-positive cells, i.e. monocytes, and cells stained yellow or pale brown were defined as NSE-negative cells. We counted 500 cells per image and, in total, 1500 cells per sample in each group. The frequencies of NSE-positive cells in each group were determined from three independent experiments.

### RT-PCR analysis

Total RNA was isolated from adherent MNCs from two groups (MC-1d, MC-5d) and the HL60 cells from three groups (control, VD, VD-TNF) using TRIZOL reagent (Invitrogen, Carlsbad, CA, USA). RT-PCR analysis was performed as previously described [29]. Briefly, 1 μg of total RNA was transcribed into first-strand cDNA using M-MLV reverse transcriptase, RNase H^−^ (Promega, Madison, WI, USA) and oligo (dT)_18_ primers, according to the manufacturer’s instructions. For detecting monocyte differentiation/undifferentiation markers (CD14, CD16, CD115/CD15), various α/β integrin chains (α1, α2, α4–6, αD, αL, αM, αX, β1, β2), Ras-related protein 1A (Rap1A), EphA (A1-A8, A10), ephrin-A (A1-A5), and GAPDH, 1 μL of the reaction mix (final 25 μL) was amplified using the reverse-transcribed cDNA as template. The mouse and human primer pairs and the cycle numbers for PCR amplification used in this study are shown in Tables 1 and 2, respectively. The RT reaction was omitted for the negative controls. The amplified mRNA expression levels were determined from three or four independent experiments and were normalized to those of GAPDH in the adherent MNCs between the two groups, to HL60 cells among the three groups, or between the control and VD-TNF groups.

**Table 1:** Primers and cycle numbers for RT-PCR amplification of mouse mRNAs

Mouse	Primer		Product size (bp)	Annealing temp. (°C)	Cycle number
CD115	Forward	5-GGGACAGCACGAGAATATAG-3’	590	53.0	26
	Reverse	5’-CACTCTGAACTGTGTAGACG-3’			
CD34	Forward	5’-ACAACCACAGACTTCCCCAA-3’	424	59.5	33
	Reverse	5’-ATTGGCCAAGACCATCAGCA-3’			
EphA1	Forward	5’-TCGAGCCTTACGCCAACTAC-3’	466	69	38
	Reverse	5’-CCGATCAGCAGAGCTATTCC-3’			
EphA2	Forward	5’-GAGTGTCCAGAGCATACCCT-3’	549	62.5	37
	Reverse	5’-GCGGTAGGTGACTTCGTACT-3’			
EpA3	Forward	5’-TGTTGGTGCTTGTGTTGCC-3’	440	57	38
	Reverse	5’-CATTTCTTGGTGCGGATGG-3’			
EphA4	Forward	5’-GGGCAGTGAATGGAGTGTCT-3’	448	61.4	38
	Reverse	5’-AGAATGACCACGAGGACCAC-3’			
EphA5	Forward	5’-CTCTGGACGTGCCTTCTC-3’	319	57	38
	Reverse	5’-TCCCCAGTCCTCCAGGAA-3’			
EphA6	Forward	5’-GTGCGGAGCTTGGCTATGTT-3’	373	61.4	38
	Reverse	5’-GAGCTGAAGGTGGTCTAGTG-3’			
EphA7	Forward	5’-ACGGGGGAAGAAACGATGTC-3’	544	61.4	38
	Reverse	5’-GCTTCCTCAAGTGTGGCAAC-3’			
EphA8	Forward	5’-TTGTGGGAGTGGAACTCGCT-3’	632	61.4	38
	Reverse	5’-CCTGTCCGTTCTGGTAATGC-3’			
EphA10	Forward	5’-ACTACCTAGAAACCGAGACC-3’	493	53.0	38
	Reverse	5’-GACACCTTGTAAAACCCTGG-3’			
ephrin-A1	Forward	5’-CATCATCTGCCCACATTACG-3’	463	60.0	37
	Reverse	5’-AGCAGTGGTAGGAGCAATAC-3’			
ephrin-A2	Forward	5’-GGTGAGCATCAACGACTACC-3’	464	61.4	38
	Reverse	5’-CTGACACTAGGAGCCCAGAA-3’			
ephrin-A3	Forward	5’-TCCGCACTACAACAGCTCAG-3’	504	62.5	38
	Reverse	5’-TAGGAGGCCAAGAGCGTCAT-3’			
ephrin-A4	Forward	5’-AAGATTCAGCGCTACACACC-3’	420	61.4	38
	Reverse	5’-GATCCTCCGACTTTGCACAT-3’			
ephrin-A5	Forward	5’-CGTGGAGATGTTGACGCTG-3’	585	57.0	38
	Reverse	5’-GGCTCGGCTGACTCATGTA-3’			
GAPDH	Forward	5’-GACTCCACTCACGGCAAAT-3’	689	57.0	22
	Reverse	5’-TCCTCAGTGTAGCCCAAGAT-3’

**Table 2:** Primers and cycle numbers for RT-PCR amplification of human mRNAs

Human	Primer		Product size (bp)	Annealing temp. (°C)	Cycle number
CD14	Forward	5’-AGAACCTTGTGAGCTGGACGAT-3’	369	61.0	24
	Reverse	5’-GAAAGTGCAAGTCCTGTGGCTT-3’			
CD15	Forward	5’-TTTGGATGAACTTCGAGTCGCC-3’	360	60.0	30
	Reverse	5’-AAGCCAGGTAGAACTTGTAGCG-3’			
CD16	Forward	5’-GACTGAAGATCTCCCAAAGG-3’	668	52.3	24
	Reverse	5’-CCTTCCAGTCTCTTGTTGAG-3’			
CD115	Forward	5’-GTGGTAGAGAGTGCCTACTT-3’	342	53.4	30
	Reverse	5’-CCATATGACGCTTACCTCTG-3’			
integrin α1	Forward	5’-GTCTATCCACGGAGAAATGG-3’	417	50.0	32
	Reverse	5’-CTCACAGAGTCCTGAAAGTC-3’			
integrin α2	Forward	5’-CTACAATGTTGGTCTCCCAG-3’	440	50.0	32
	Reverse	5’-CAACATCTATGAGGGAAGGG-3’			
integrin α4	Forward	5’-GATCATCTTACTGGACTGGC-3’	320	54.8	32
	Reverse	5’-CAGATCTGAGAAGCCATCTG-3’			
integrin α5	Forward	5’-CCAGCCCTACATTATCAGAG-3’	390	50.0	32
	Reverse	5’-GAGATGAGGGACTGTAAACC-3’			
integrin α6	Forward	5’-GGAGATAAACTCCCTGAACC-3’	416	50.0	32
	Reverse	5’-CGAGAATAGCCACTAGGATG-3’			
integrin αD	Forward	5’-ATCAGCAGGCAGGAAGAATC-3’	518	50.1	32
	Reverse	5’-ACCTCGTCTTCTTCTAGCAC-3’			
integrin αL	Forward	5’-GCCCTGGTTTTCAGGAATG-3’	430	53.0	32
	Reverse	5’-CCAATCCCGATGATGTAGC-3’			
integrin αM	Forward	5’-GTGTGATGCTGTTCTCTACG-3’	364	50	32
	Reverse	5’-CTCCATGATTGCCTTGACTC-3’			
integrin αX	Forward	5’-CCAGATCACCTTCTTGGCTAC-3’	523	61.3	32
	Reverse	5’-CTTCAGGGTGAAATCCAGCTC-3’			
integrin β1	Forward	5’-TTCAAGGGCAAACGTGTGAG-3’	459	54.4	28
	Reverse	5’-CCGTGTCCCATTTGGCATTC-3’			
integrin β2	Forward	5’-CAATAAACTCTCCTCCAGGG-3’	522	52.5	28
	Reverse	5’-CAGTACTGCCCGTATATCAG-3’			
Rap1A	Forward	5’-GTACAAGCTAGTGGTCCTTG-3’	371	50.0	29
	Reverse	5’-CAACTACTCGCTCATCTTCC-3’			
EphA1	Forward	5’-TGGTGGAACTTCCTTCGAGA-3’	446	61.2	35
	Reverse	5’-ACAATCCCAAAGCTCCACAC-3’			
EphA2	Forward	5’-CGCCGGCTCTGATGCACCTT-3’	312	62.0	38
	Reverse	5’-TCCTGAGGTGCCCGGAAGAA-3’			
EphA3	Forward	5’-TGCGGTCAGCATCACAACTA-3’	380	59.3	38
	Reverse	5’-TTGCTACTGCCGCTGAAATG-3’			
EphA4	Forward	5’-TGCAGCTTTTGTCATCAGCC-3’	381	60.3	38
	Reverse	5’-TTAGTGACCACGCCTTCCAA-3’			
EphA5	Forward	5’-CTCTGGACGTGCCTTCTC-3’	319	56.6	38
	Reverse	5’-TCCCCAGTCCTCCAGGAA-3’			
EphA6	Forward	5’-TGCGAAGTCCGGGAATTTCT-3’	627	61.6	38
	Reverse	5’-ACGAACAGTGAAGGGGCATT-3’			
EphA7	Forward	5’-AAAGCTGACCAAGAAGGCGA-3’	311	62.0	38
	Reverse	5’-TCAAACTGCCCCATGATGCT-3’			
EphA8	Forward	5’-ATCACCTACAATGCCGTGTG-3’	341	58.4	38
	Reverse	5’-TACTCCAGGATGATGCCGTT-3’			
EphA10	Forward	5’-CACGTACCAAGTGTGCAATG-3’	646	56.8	38
	Reverse	5’-CAACTGGATACCTTCGCAGA-3’			
ephrin-A1	Forward	5’-GGAACCCAGACCCATAGGAG-3’	825	59.7	38
	Reverse	5’-CCCGTTTTGAGGCTGCTAGG-3’			
ephrin-A2	Forward	5’-TACGTGCTGTACATGGTCAACG-3’	316	58.1	38
	Reverse	5’-GGCTGCTACACGAGTTATTGCT-3’			
ephrin-A3	Forward	5’-TTTACTGCCCGCACTACAACAG-3’	523	59.7	38
	Reverse	5’-AACGTCATGAGGAAGAAGGCGA-3’			
ephrin-A4	Forward	5’-GCCCCGAGACGTTTGCTTTGTA-3’	364	62.5	34
	Reverse	5’-TTGTCGGTCTGAATTGGCACCC-3’			
ephrin-A5	Forward	5’-ACTCTCCAAATGGACCGCTGAA-3’	363	66.0	38
	Reverse	5’-TCAAAAGCATCGCCAGGAGGAA-3’			
GAPDH	Forward	5’-GTCGGAGTCAACGGATTTGG-3’	607	57.2	21
	Reverse	5’-GGATGATGTTCTGGAGAGCC-3’			

### Flow cytometry

We examined cell surface expression of α4, α5, α6, αL, αM, αX, β1, and β2 integrin subunit proteins by flow cytometry because they were clearly expressed, as detected by the RT-PCR analysis in HL60 cells of the control and VD-TNF groups. Cells were prepared at a concentration of 1 × 10^6^ /50 μL in PBS containing 1% bovine serum albumin (BSA; Fraction V, Sigma-Aldrich) and 2 mM EDTA. To avoid non-specific Fc-gamma receptor-mediated binding of fluorochrome-conjugated antibodies, cell suspensions were pretreated with 20 μL of the human Fc receptor-binding inhibitor (20 μL/10^6^ cells; Affymetrix, San Diego, CA, USA) for 20 min at 4 °C according to the manufacturer’s instructions. To the cell suspensions, we added FITC-conjugated anti-α4 antibody (5 μL/10^6^ cells; Miltenyi Biotec, Bergisch Gladbach, Germany), PE-conjugated anti-α5 antibody (5 μL/10^6^ cells; Miltenyi Biotec), PE-conjugated anti-α6 antibody (5 μL/10^6^ cells; Miltenyi Biotec), FITC-conjugated anti-αL antibody (5 μL/10^6^ cells; Miltenyi Biotec), FITC-conjugated anti-αM antibody (0.25 μg /10^6^ cells; Tonbo Biosciences, San Diego, CA, USA), APC-conjugated anti-αX antibody (20 μL/10^6^ cells; BD Biosciences, San Jose, CA, USA), FITC-conjugated anti-ß1 antibody (5 μL/10^6^ cells; Miltenyi Biotec), or APC-conjugated anti-ß2 antibody (5 μL/10^6^ cells; Miltenyi Biotec), and then incubated the cells for 20 min at room temperature. After washing, 50,000 cells were analyzed for their expression characteristics by using a flow cytometer (S3 Cell Sorter; Bio-Rad Laboratories, Hercules, CA, USA). We used cell suspensions pretreated with the human Fc receptor-binding inhibitor as controls. Some of the cell suspensions pretreated with the human Fc receptor-binding inhibitor were treated with an isotype control antibody (APC-conjugated mouse IgG1, 0.5 μg/10^6^ cells; Tonbo).

### Cell adhesion stripe assay and time-lapse microscopy

Because treatment with VD and TNF was most effective for monocytic differentiation of HL60 cells, we examined the adhesion of HL60 cells in the VD-TNF group to a Matrigel-coated coverslip surface on which EphA2-Fc or ephrin-A1-Fc was adsorbed in stripes according to the method described by Ogawa et al. [30] with some modifications. Briefly, coverslips (15 mm in diameter) were incubated for 3 h in 100 μg/mL poly-L-ornithine (Sigma-Aldrich) in PBS, washed with sterile water, and dried. Comb-shaped silicon masks with parallel teeth and gaps of about 0.48 mm width were then applied to the glass surface. EphA2-Fc (100 μL of 8 μg/mL in HBSS; R&D Systems, Minneapolis, MN, USA), ephrin-A1-Fc (R&D Systems), or human IgG Fc as a control (Fc; OEM Concepts, Inc., Toms River, NJ, USA) was then adsorbed onto the surface for 60 min. The coverslips were washed with HBSS and the masks were removed. After washing, Fc (100 μL of 8 μg/mL in HBSS) followed by Matrigel (100 μL of 40 μg/mL in HBSS; Corning Inc., Tewksbury, MA, USA) was adsorbed on the surface for 60 min. After washing, the coverslips were placed in 6 cm culture dishes with 5 mL RPMI 1640 containing 2% FBS. Cells were plated at a density of 3 × 10^5^ cells/mL and were allowed to adhere for 16 h at 37 °C. Cells were then fixed in 4% paraformaldehyde in PBS for 15 min at room temperature. Phase-contrast images of the fields including both the Fc-chimera protein-adsorbed (test) and the adjacent Fc and Matrigel-adsorbed control regions were acquired using a 4 × and 10 × objective lens (IX71; Olympus). For quantitative analysis of cell density in the test versus the control regions, we counted cell numbers in an area of 0.48 mm × 0.96 mm in each of the test and control regions. This was repeated for each Fc-chimera protein tested. Cell densities were determined from three independent experiments and were normalized to those from the Fc plus Matrigel-only adsorbed control region.

Cell adhesion behaviors on coverslips coated with the EphA2-Fc or with ephrin-A1-Fc in stripes were also analyzed by time-lapse microscopy as previously described [29] with minor modifications. HL60 cells in the VD-TNF group at a density of 3 × 10^5^ cells/mL (1.5 mL/3.5-cm culture dish) were plated on coverslips coated with EphA2-Fc or ephrin-A1-Fc in stripes and placed in a 3.5-cm culture dish in an incubator (maintained at 37 °C in humidified 5% CO_2_/95% air; ONI-INU-F1, Tokai Hit Co., Ltd., Fujinomiya, Japan) installed on the stage of an inverted microscope (IX71, Olympus). Phase contrast images were obtained at 2 min intervals (for 16 h) using a digital camera (DP72, Olympus) controlled by the manufacturer’s software (DP2-BSW, Olympus).

### Visualization of focal adhesions and F-actin

We examined the formation of focal adhesions and F-actin in the cells after incubation for 16 h on coverslips coated with EphA2-Fc or ephrin-A1-Fc in stripes, as described in the cell adhesion stripe assay, by fluorescence microscopy according to the method of Ogawa et al. [29] with some modifications. Briefly, HL60 cells from the VD-TNF group on the coverslips were fixed with 4% paraformaldehyde in PBS for 15 min at room temperature. The cells were then incubated with 0.02% Triton X-100 in PBS for 15 min at room temperature. To visualize focal adhesions, cells were pre-incubated with 1% BSA in PBS for 30 min in a humidified chamber, followed by incubation with an anti-human vinculin monoclonal antibody (hVIN-1, Sigma-Aldrich) at a dilution of 1:200 in 1% BSA-PBS for 60 min at 32 °C. After washing with PBS, the cells were incubated with a mixture of Alexa 488-conjugated goat anti-mouse IgG (5 μg/mL; Molecular Probes, Inc., Eugene, OR, USA) and 165 nM Alexa 568-labeled phalloidin (to visualize F-actin; Invitrogen) in 1% BSA-PBS for 30 min at 32 °C, followed by washing with PBS and mounting with PermaFluor (Thermo Fisher Scientific, Fremont, CA, USA). The cells were photographed under a fluorescence microscope (IX71; Olympus).

### Statistical analysis

Statistical analyses were performed with the statistical software package Statcel 2 (OMS Publishing Inc., Tokorozawa, Japan). The bar graphs represent means ± SD. Unpaired *t*-test was used to determine the statistical significance of the results. *P* values less than 0.05 were considered significant.

## Results

### EphA and ephrin-A are upregulated in bone marrow mononuclear cells during monocytic maturation

**Fig. 1:**
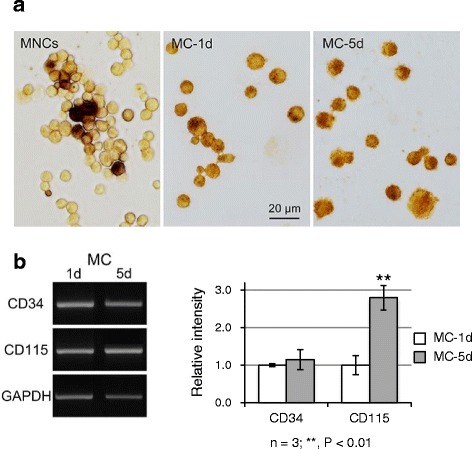
M-CSF induces differentiation of non-adherent bone marrow MNCs to adherent cells of the monocytic lineage. **a** NSE staining of mouse bone marrow MNCs immediately after fractionation and of adherent MNCs from the MC-1d group (non-adherent MNCs cultured in medium containing 20 ng/mL M-CSF that adhered within one day) and MC-5d group (non-adherent MNCs cultured in medium containing 20 ng/mL M-CSF that adhered within two days and were cultured for three more days). **b** RT-PCR amplification of the myeloid cell marker CD34, and the monocyte marker CD115 in bone marrow MNCs from the MC-1d and MC-5d group. Densitometric quantification of mRNA expression levels from three independent experiments, normalized to GAPDH, is shown as the means ± SD. The expression of CD115 in the MC-5d group is upregulated 2.80-fold (*P* = 0.002) compared to that in the MC-1d group, whereas CD34 expression levels are almost the same between both groups

M-CSF induces proliferation and differentiation of bone marrow MNCs into the mononuclear phagocytic lineage, wherein the M-CSF receptor signaling is involved in cell adhesion to extracellular matrices [31]. Bone marrow MNCs, immediately after fractionation by equilibrium density centrifugation, consisted of many non-adherent/NSE-negative cells and some adherent/NSE-positive cells (Fig. 1a). Non-adherent/NSE-negative MNCs were selected and differentiated to monocytes by treatment with M-CSF to examine the expression levels of EphA and ephrin-A during the monocyte maturation process. First, we examined NSE-reactivity and the mRNA expression levels of marker molecules (CD115, a monocyte marker [23, 24], CD34, a myeloid cell marker [25]) in adherent MNCs by semi-quantitative RT-PCR in both groups (MC-1d group: non-adherent MNCs cultured in M-CSF-containing medium which became adherent in one day; MC-5d group: non-adherent MNCs cultured in M-CSF-containing medium which became adherent in two days and were cultured for three more days). All cells from both groups became NSE-positive (Fig. 1a), and the expression level of CD115 in the MC-5d group was elevated 2.80-fold and significantly higher than that in the MC-1d group (*P =* 0.002), whereas the expression levels of CD34 were similar between both groups (Fig. 1b). These findings indicate that adherent MNCs in these two groups likely belong to the monocyte lineage undergoing maturation and that the cells from the MC-5d group are closer to mature monocytes.

**Fig. 2:**
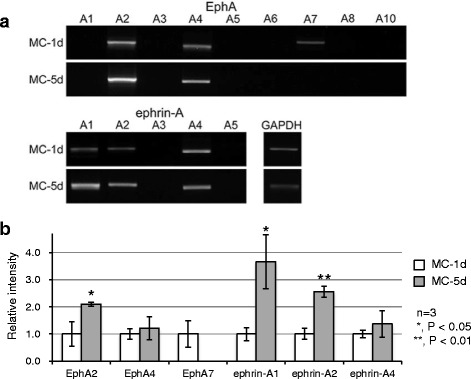
RT-PCR amplification of EphA and ephrin-A mRNAs from mouse bone marrow MNCs treated with M-CSF. **a** MC-1d represents non-adherent MNCs cultured in medium containing 20 ng/mL M-CSF that adhered within one day, and MC-5d represents non-adherent MNCs cultured in medium containing 20 ng/mL M-CSF that adhered in two days and were cultured for three more days. Compared to other EphAs and ephrin-As, EphA2 and ephrin-A1 required one less cycle of amplification. **b** Densitometric quantification of mRNA expression levels from three independent experiments, normalized to GAPDH, is shown as the mean ± SD. The expression levels of EphA2, ephrin-A1, and ephrin-A2 in the MC-5d group were upregulated by 2.10, 3.66, and 2.56-fold, respectively (*P =* 0.014, *P =* 0.011, *P =* 0.001) compared to that in the MC-1d group, whereas EphA7 expression was downregulated in the MC-5d group and was less than the detection limit after 38 cycles of PCR amplification

We screened EphA and ephrin-A mRNA expression in the adherent MNCs of two groups by RT-PCR to examine whether these molecules were up- or downregulated during monocytic maturation. EphA2, EphA4, ephrin-A1, ephrin-A2, and ephrin-A4 were detected in the adherent MNCs of both groups. EphA2 and ephrin-A1 expression was distinctly higher among the EphA and ephrin-A members compared to that of other receptors and ligands, respectively, as observed by stronger band intensities despite one less cycle of PCR amplification (Fig. 2a). EphA7 was detected in the MC-1d group but was decreased to a level less than the detection threshold of PCR amplification in the MC-5d group. This indicates that EphA7 expression was likely downregulated during monocyte maturation. We compared mRNA expression in adherent MNCs between the two groups. The expression levels of EphA2, ephrin-A1, and ephrin-A2 in the MC-5d group were 2.10, 3.66, and 2.56-fold higher, respectively, and were significantly increased compared to those in the MC-1d group (*P =* 0.014, *P =* 0.011, *P =* 0.001; Fig. 2b). There were no significant differences in the expression levels of EphA4 and ephrin-A4 between the two groups, although they tended to increase in the MC-5d group (Fig. 2b).

### Treatment with VD plus TNFα efficiently differentiates HL60 cells into monocytes

**Fig. 3:**
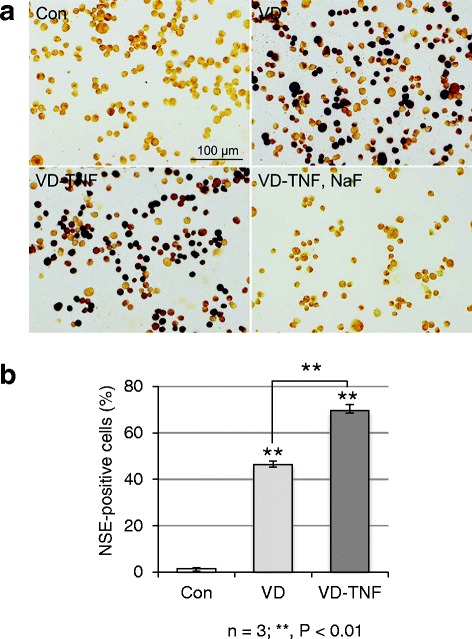
Treatment with a combination of VD and TNFα effectively induces monocyte differentiation in HL60 cells. **a** NSE staining of HL60 cells treated with the vehicle (control group; Con), 50 nM VD (VD group; VD), and 50 nM VD with 5 ng/mL TNFα (VD-TNF group; VD-TNF). Note that NSE-reactivities in the VD-TNF group become negative by adding 40 mM fluoride (NaF) to the reaction medium, indicating that NSE-positive cells are possibly monocytes. **b** Frequencies of NSE-positive cells in the control, VD, and VD-TNF groups as determined from three independent experiments. Cells stained brown to dark brown or almost black were defined as NSE-positive cells. At random, three field images per smear sample, 500 cells per image, and thus 1500 cells per sample were counted in each group. NSE-positive cells were 46.4 ± 1.5% and 69.5 ± 2.7% in the VD and VD-TNF group, respectively, and are significantly increased compared to those in the control group (1.5 ± 0.4%). The frequency of NSE-positive cells in the VD-TNF group is 1.50-fold greater and statistically significant compared to that in the VD group. *P < 0.01

HL60 cells have been widely used as a terminal differentiation model of monocytes, and VD and TNFα are used to induce monocytic differentiation [17–19]. HL60 cells adhered to tissue culture dishes and frequently ceased cell propagation in the VD group (HL60 cells cultured in VD-containing medium for 3 days), and this was more prominent in the VD-TNF group (HL60 cells cultured in VD-containing medium for 2 days and thereafter in VD plus TNFα for 1 day). Thus, we determined the differentiation efficiencies of HL60 to monocytes in both groups cytochemically, by examining the fluoride-sensitive NSE activity that is specific for monocytes/macrophages. NSE-reactivity showed various color ranges (brown to dark brown or almost black) in the NSE-positive cells of the VD and VD-TNF groups (Fig. 3a). NSE activity in these cells was almost completely inhibited by the addition of 40 mM fluoride to the reaction medium, and the colors turned to yellow or pale brown in almost all cells. In contrast, almost all cells in the control group stained pale brown or yellow and were thus considered NSE-negative. Next, we compared the frequency of NSE-positive cells among the three groups. In the control group, the frequency of the NSE-positive cells that stained light brown in most cases was 1.5 ± 0.4% of the total (Fig. 3b). In the VD and VD-TNF group, the frequencies of NSE-positive cells that were dark brown or almost black in many cases were 46.4 ± 1.5% and 69.5 ± 2.7%, respectively, and were significantly increased compared to those in the control. The frequency of the NSE-positive cells in the VD-TNF group was increased by 1.50-fold and was significantly higher than that in the VD group (*P <* 0.001).

**Fig. 4:**
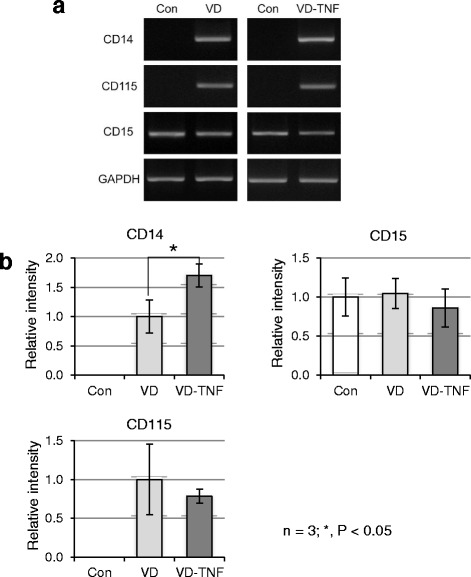
RT-PCR amplification of monocyte markers and a myeloid cell marker in HL60 cells. **a** Treatment with VD and/or TNFα induces monocyte markers CD14 and CD115 expression in HL60 cells. **b** Densitometric quantification of mRNA expression levels from three independent experiments, normalized to GAPDH, is shown as means ± SD. The expression level of CD14 in the VD-TNF group is 1.71-fold and significantly higher than that in the VD group (*P =* 0.024) whereas the expression levels of CD115 are similar between both groups

We then examined the mRNA expression levels of marker molecules (CD14, CD16, and CD115, monocyte markers [5, 23, 24]; CD15, a myeloid cell marker [26]) in HL60 cells from the three groups by semi-quantitative RT-PCR. CD14, CD16, and CD115 expression was not detected in the control group. CD16 was not clearly expressed in our RT-PCR analysis even with 30 amplification cycles, whereas CD14 and CD115 were readily detected in the VD and VD-TNF group (Fig. 4a). The expression level of CD14 in the VD-TNF group was increased 1.71-fold, significantly higher than that in the VD group (*P =* 0.024), whereas the expression level of CD115 was similar in the two groups (Fig. 4b). CD15 mRNA was detected in all groups, and its expression level was similar among all three groups (Fig. 4b). NSE staining and RT-PCR analyses showed that treatment with the combination of VD and TNFα was more effective for differentiation of HL60 into monocytes, which could be considered as classical monocytes (CD14^++^CD16^−^) [24]. Therefore, we used HL60 cells from the VD-TNF group for further experiments.

### EphA2, EphA4, and ephrin-A4 are induced and/or upregulated during differentiation of HL60 cells into monocytes

**Fig. 5:**
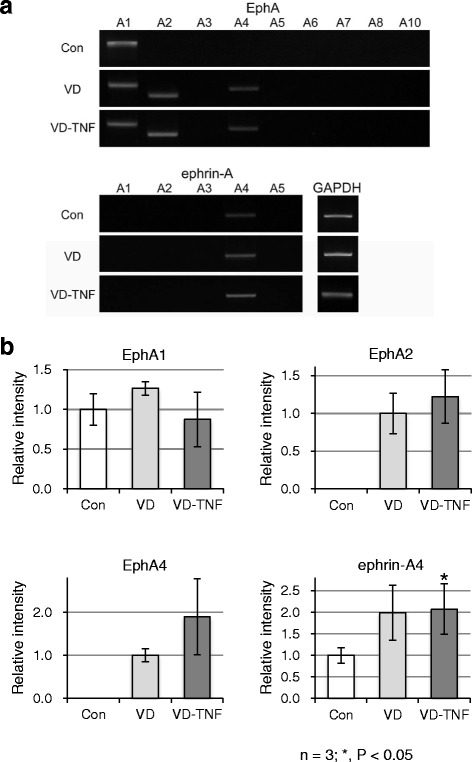
RT-PCR amplification of EphA and ephrin-A mRNAs in HL60 cells. HL60 cells were treated with the vehicle (control group; Con), 50 nM VD (VD group; VD), and 50 nM VD with 5 ng/mL TNFα (VD-TNF group; VD-TNF). **a** Treatments with VD and/or TNFα induce EphA2 and EphA4 expression in HL60 cells. **b** Densitometric quantification of mRNA expression levels from three independent experiments, normalized to GAPDH, is shown as the mean ± SD. The expression level of EphA1 was similar among the three groups whereas that of ephrin-A4 in the VD-TNF group was elevated 2.08-fold and was significantly higher than that in the control (*P =* 0.039)

We screened the mRNA expression of all members of the EphA and ephrin-A groups in HL60 cells from the three groups by RT-PCR to determine whether these receptors and ligands were expressed, and if they were upregulated or downregulated during monocytic differentiation. Among the EphA receptors and ephrin-A ligands, EphA1 and ephrin-A4 were detected in HL60 cells from the control group, whereas EphA1, EphA2, EphA4, and ephrin-A4 were detected in HL60 cells from the VD and VD-TNF groups (Fig. 5a). This indicates that EphA2 and EphA4 expression is likely induced during monocytic differentiation.

We compared the expression of these mRNAs in HL60 cells among the three groups. The expression of EphA1 was similar among the three groups (Fig. 5b). The expression levels of EphA2 and EphA4 induced in the VD and VD-TNF group were not significantly different between the two groups (*P =* 0.491, *P =* 0.160) although expression levels of both receptors in the VD-TNF group tended to increase slightly compared to those in the VD group. The expression of ephrin-A4 in the VD-TNF group was elevated 2.08-fold and was significantly higher than that in the control (*P =* 0.039) whereas that in the VD group was not statistically different (*P =* 0.060) compared to that in the control (Fig. 5b). The overall expression patterns of EphA/ephrin-A were similar between the VD and VD-TNF groups. These results show that EphA2, EphA4, and ephrin-A4 expression are induced and/or upregulated during monocytic differentiation in HL60 cells whereas naïve cells express substantial amounts of EphA1 and ephrin-A4.

### Integrin expression during monocytic differentiation of HL60 cells

Before examining the adhesive behavior of HL60 cells, we screened for the expression of various α and β integrin subunits (α1, α2, α4–6, αD, αL, αM, αX, β1, β2), and Rap1A in HL60 cells from the control and VD-TNF groups by RT-PCR. These integrins are generally expressed in leukocytes [4, 32], and Rap1A, a small GTPase, is an important regulator of conformational changes in integrins to the high affinity form for generating integrin ligands via external stimuli such as chemokines [5–7].

**Fig. 6:**
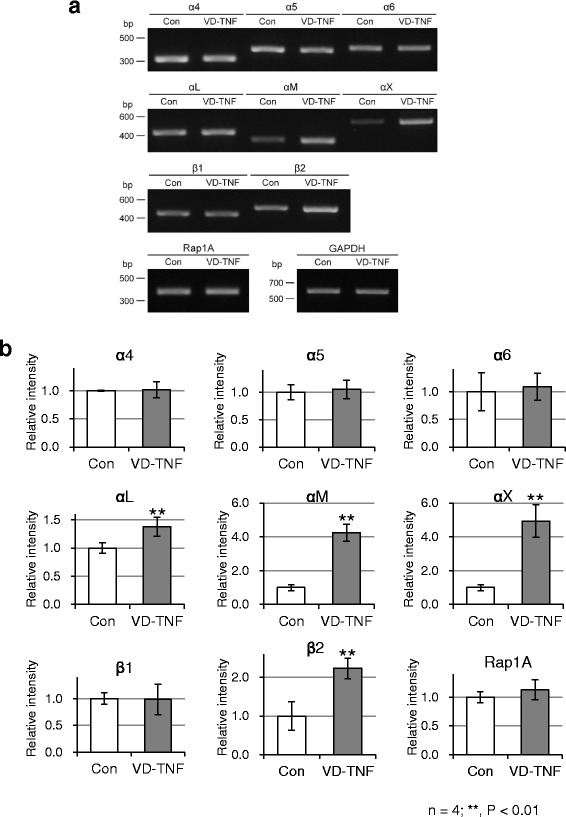
RT-PCR amplification of integrin subunits and Rap1A mRNAs in HL60 cells. RT-PCR amplification (**a**) and densitometric quantification (**b**) of α4, α5, α6, αL, αM, αX, β1, and β2 integrin subunits as well as Rap1A expression in HL60 cells treated with the vehicle (Con) and 50 nM VD plus 5 ng/mL TNFα (VD-TNF). Data from four independent experiments, normalized to GAPDH, are shown as means ± SD. HL60 cells in the control group likely express relatively large amounts of α4β1, α5β1, and α6β1, αLβ2 integrins and substantial amounts of αMβ2 and αXβ2 integrins, whereas HL60 cells in the VD-TNF group likely express relatively large amounts α4β1, α5β1, α6β1, αLβ2, αMβ2, and αXβ2 integrins. *p < 0.05, **p < 0.01

**Fig. 7:**
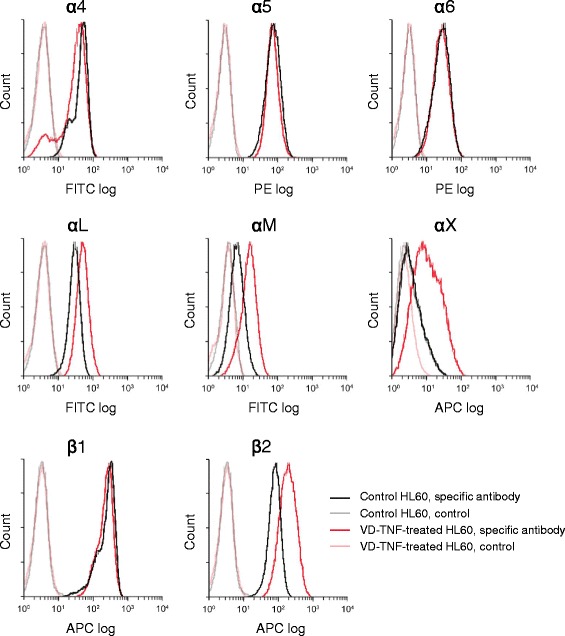
Cell surface expression of integrin subunit proteins in HL60 cells. Representative histograms from flow cytometric analyses, showing the cell surface expression of α4, α5, α6, αL, αM, αX, β1, and β2 integrin subunits in HL60 cells of the control group (black line, specific antibody; gray line, control) and in the VD-TNF group (red line, specific antibody; pink line, control). For the control in each group, cell suspensions were pretreated with the human Fc receptor-binding inhibitor and with an APC-conjugated isotype control antibody in case of the APC-conjugated anti-αX, anti-β1, and anti-β2 antibody. All integrin subunits are clearly expressed except for the αX integrin subunit in HL60 cells of the control group

The α1, α2, and αD integrin subunits were not clearly expressed in HL60 cells in our RT-PCR analysis up to 32 amplification cycles although the other examined integrin subunits were detected in 28 or 32 amplification cycles in HL60 cells from both groups. Relatively high expression of α4, α5, α6, αL, β1, and β2 integrin subunits was detected in HL60 cells of the control group. In contrast, high expression of α4, α5, α6, αL, αM, αX, β1, and β2 integrin subunits was detected in HL60 cells from the VD-TNF group (Fig. 6a). The expression levels for α4, α5, α6, and β1 subunits were almost the same between the control and VD-TNF group. In contrast, the expression levels of αL, αM, αX, and β2 subunits in the VD-TNF group were 1.38 ± 0.17, 4.25 ± 0.50, 4.93 ± 0.97, and 2.23 ± 0.27 fold higher than those in the control group, respectively (*P* = 0.008, *P* < 0.001, *P* = 0.004, *P* = 0.002; Fig. 6b). Moreover, high expression of Rap1A was detected in HL60 cells of both the control and VD-TNF groups. We also examined cell surface expression of α4, α5, α6, αL, αM, αX, β1, and β2 integrin subunit proteins in HL60 cells of the control and VD-TNF group by flow cytometry. Expression levels of α4, α5, α6, and β1 subunits in HL60 cells were almost the same between the control and VD-TNF group (Fig. 7). In contrast, expression levels of αL, αM, αX, and β2 subunits in the VD-TNF group were obviously higher compared to those in the control, while the αX subunit was not clearly detected in HL60 cells of the control (Fig. 7). Thus relative expression levels of the cell surface proteins tested by flow cytometry were nearly identical to those of the mRNA by RT-PCR in each integrin subunit in HL60 cells between the control and VD-TNF group.

These data indicated that naïve HL60 cells likely express relatively large amounts of α4β1 (CD49d/CD29, VLA-4), α5β1, α6β1 and αLβ2 (CD11a/CD18, LFA-1) integrins with substantial levels of αMβ2 (CD11b/CD18, Mac-1) integrin. In contrast, HL60 monocytes likely express relatively large amounts of α4β1, α5β1, α6β1, αLβ2, αMβ2, and αXβ2 (CD11c/CD18) integrins. Moreover, the integrin regulator Rap1A is expressed in HL60 cells from both groups. These results indicated that HL60 monocytes as well as naïve HL60 cells likely possess sufficient adhesive ability towards integrin ligands, including membrane proteins such as ICAM-1 and VCAM-1, and extracellular matrix proteins, including laminin and collagen, based on the ligand specificities of the expressed integrins (α4β1: VCAM-1, MAdCAM-1, etc.; α5β1: fibronectin, osteopontin, etc.; α6β1: laminin, thrombospondin, etc.; αLβ2, αMβ2 and αXβ2: ICAM-1, collagen, etc.) [4, 33].

### EphA and ephrin-A activation likely promotes cell adhesion and formation of cytoplasmic protrusions in HL60 monocytes

**Fig. 8:**
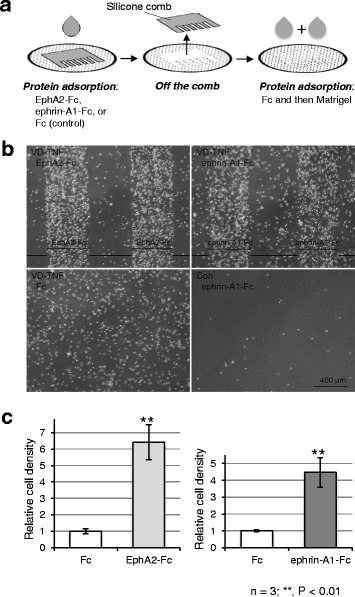
HL60 cells treated with VD and TNFα preferentially occupy the EphA2-Fc or ephrin-A1-Fc-adsorbed surface. **a** A schematic drawing illustrating the procedure of Fc-chimera protein adsorption to the coverslip surface in stripes. A comb-shaped silicon mask was applied to the coverslip surface. The Fc-chimera or Fc proteins were adsorbed onto the surface. After washing, the mask was removed. Subsequently, Fc followed by Matrigel was adsorbed onto the glass surface. After washing, the coverslips were placed in 6 cm culture dishes with culture medium. HL60 cells were plated and cultured for 16 h. **b** Typical phase-contrast micrographs showing HL60 cells treated with VD and TNFα (VD-TNF group) cultured on the coverslip surface wherein regions adsorbed with EphA2-Fc or ephrin-A1-Fc appeared as stripes. In the control stripes using Fc instead of the chimera proteins, HL60 cells of the VD-TNF group did not form stripes with different cell densities. Native HL60 cells (Con) scarcely adhere to an ephrin-A1-Fc-adsorbed surface. **c** Quantified cell densities in regions adsorbed with EphA2-Fc or ephrin-A1-Fc and Fc plus Matrigel compared to those with Fc and Matrigel in HL60 cells from the VD-TNF group. The results from three independent experiments are shown. Data are presented as means ± SD. **P < 0.01

To examine whether EphA/ephrin-A activation affects integrin-mediated cell adhesion to the extracellular matrix surface in HL60 cells during monocytic differentiation, we compared adhesive properties between HL60 cells of the control and the VD-TNF groups on coverslip surfaces to which EphA2-Fc or ephrin-A1-Fc was adsorbed in stripes and Fc plus Matrigel was overlaid. A test region of the Fc-chimera protein adsorbed surface presented in stripes at certain intervals on the Fc plus Matrigel-adsorbed coverslip (Fig. 8a). Using phase-contrast microscopy, we found that HL60 cells from the VD-TNF group formed stripes with different cell densities corresponding to the stripes of the Fc-chimera protein-absorbed surface. They preferentially occupied ephrin-A1-Fc-adsorbed as well as EphA2-Fc-adsorbed test surfaces rather than only the Fc plus Matrigel-adsorbed control surface (Fig. 8b). In contrast, HL60 cells of the VD-TNF group were adhered to the surface of control coverslips on which Fc was adsorbed instead of the Fc-chimera protein, but did not form stripes of different cell densities (Fig. 8b). Moreover, HL60 cells of the control group scarcely adhered to the Fc-chimera protein-adsorbed test coverslips or the control coverslips. We calculated the cell densities of HL60 cells from the VD-TNF group on the Fc-chimera protein-adsorbed region relative to those on the adjacent control region. The relative cell densities on EphA2-Fc and ephrin-A1-Fc-adsorbed regions compared to the control regions were increased 6.4 ± 1.1 and 4.5 ± 0.9 fold (means ± SD), respectively, and showed significant differences from the controls (*P <* 0.001, *P =* 0.002; Fig. 8c). These results, along with the RT-PCR findings, indicate that EphA2 and/or EphA4 activation induced by ephrin-A1-Fc as well as ephrin-A4 activation induced by EphA2-Fc in HL60 monocytes, likely promote cell adhesion to Matrigel, which contains large amounts of integrin ligands, mainly laminin and type IV collagen. Moreover, adhesiveness to Matrigel and its potentiation by ephrin-A and EphA activation likely appears during monocytic differentiation in HL60 cells, although naïve HL60 cells also express substantial amounts of EphA1, ephrin-A4, the integrins targeting the extracellular matrices, as well as Rap1A.

**Fig. 9:**
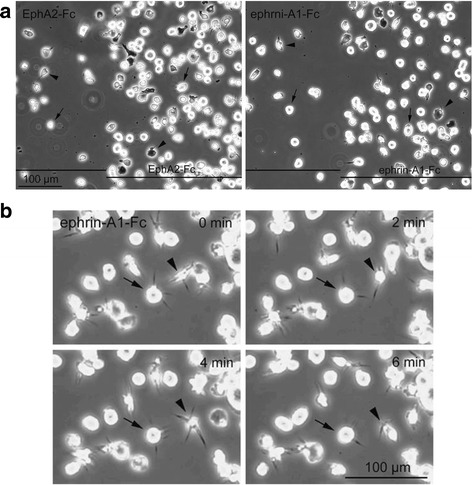
Typical micrographs of HL60 cells from the VD-TNF group on EphA2-Fc or ephrin-A1-Fc-adsorbed surfaces. **a** HL60 cells from the VD-TNF group were cultured on a coverslip surface on which the regions adsorbed with Fc, EphA2-Fc or ephrin-A1-Fc and Fc plus Matrigel and those with Fc plus Matrigel appeared alternatively as stripes, and phase-contrast images were photographed. Bright/round (arrows) and dark/polymorphic cells (arrowheads) are present as the major and minor populations of cells, respectively, observed not only on the EphA2-Fc or ephrin-A1-Fc-adsorbed test surfaces but also on the control surface. **b** A series of time-lapse micrographs obtained at 2-min intervals in the ephrin-A1-Fc-adsorbed region showing the extension and retraction of a number of cytoplasmic protrusions from the cells (arrows and arrowheads)



Under phase-contrast microscopy, two types of cells were observed on the EphA2-Fc or ephrin-A1-Fc and Fc plus Matrigel-adsorbed test surfaces, and on the Fc plus Matrigel-adsorbed control surface. The majority of cells were bright and round and a minor proportion of cells was observed to be dark and polymorphic, i.e., spread on the surfaces to some extent (Fig. 9a). Therefore, we then examined the adhesive behavior of HL60 cells from the VD-TNF group on EphA2-Fc or ephrin-A1-Fc and the integrin ligand-adsorbed surfaces by time-lapse microscopy, wherein phase-contrast images were obtained at 2 min intervals for 16 h. Cells were found to behave similarly on both the EphA2-Fc and ephrin-A1-Fc-adsorbed test surfaces. Cell density differences between the two regions, corresponding to the stripes of the Fc-chimera proteins, formed quickly within a few minutes after seeding and remained largely unchanged afterwards as observed by time-lapse microscopy. At a few hours after seeding and thereafter, many cells on the Fc plus Matrigel-adsorbed control regions moved relatively rapidly whereas the cells on the adjacent EphA2-Fc or ephrin-A1-Fc and Fc plus Matrigel-adsorbed test regions were mainly confined to a narrow area. In other words, when the cells moved from the control region to the adjacent test region, most cells abruptly stopped, temporarily spread, and remained in the restricted narrow area as if they were attached or tethered to these surfaces (see Additional file 1: Video S1 and Additional file 2: Video S2). Bright round cells that remained in a narrow area on the test surfaces frequently repeated the extension and retraction of cytoplasmic protrusions towards every direction in the X-Y plane (Fig. 9b). These results indicated that ephrin-A4 activation by EphA2-Fc and EphA2, and/or EphA4 activation by ephrin-A1-Fc likely promotes cell adhesion and induces extension and retraction of cytoplasmic protrusions in HL60 monocytes.

**Additional file 1:** **Video S1.** Cell adhesion behaviors on coverslips coated with EphA2-Fc in stripes. (MOV 7290 kb)

**Additional file 2:** **Video S2.** Cell adhesion behaviors on coverslips coated with ephrin-A1-Fc in stripes. HL60 cells in the VD-TNF group at a density of 3 × 10^5^ cells/mL (1.5 mL/3.5-cm culture dish) were plated on coverslips coated with EphA2-Fc or ephrin-A1-Fc in stripes and Fc plus Matrigel overall, and placed in a 3.5 cm culture dish in an incubator (maintained at 37 °C in humidified 5% CO_2_/95% air; ONI-INU-F1, Tokai Hit Co., Ltd., Fujinomiya, Japan) installed on the stage of an inverted microscope (IX71, Olympus). Phase contrast images were obtained at 2 min intervals (for 16 h) using a digital camera (DP72, Olympus) controlled by software (DP2-BSW, Olympus). Videos starting from a few min after cell seeding consist of a series of time-lapse micrographs in a field wherein a region adsorbed with EphA2-Fc (Additional file 1: Video S1) or ephirn-A1-Fc (Additional file 2: Video S2) and Fc plus Matrigel (left side) and that with Fc plus Matrigel (right side) appears adjacently. (MOV 7230 kb)


### EphA and ephrin-A activation likely promote focal adhesion formation in HL60 monocytes

**Fig. 10:**
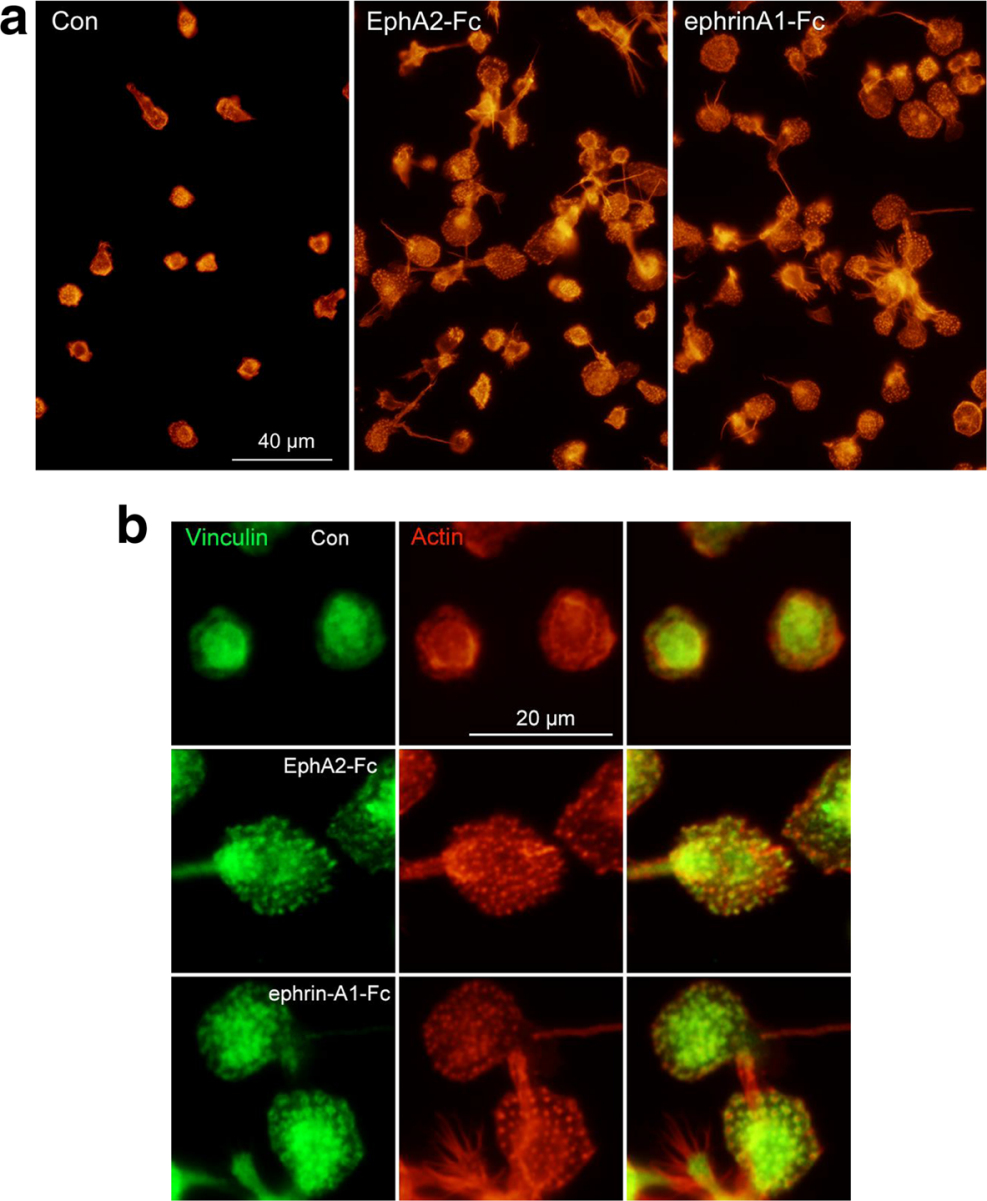
Representative images of F-actin and vinculin highlighting HL60 cell morphology in the VD-TNF group. HL60 cells from the VD-TNF group were cultured on a coverslip surface on which the regions adsorbed with EphA2-Fc or ephrin-A1-Fc and Fc plus Matrigel and those with Fc plus Matrigel appeared alternatively as stripes. Fluorescence images of F-actin staining (red) and vinculin immunostaining (green) highlighting morphology of cells on the Fc and Matrigel-adsorbed control surface (Con), the EphA2-Fc and Fc plus Matrigel-adsorbed, and the ephrin-A1-Fc and Fc plus Matrigel-adsorbed test surfaces were photographed. **a** Cells extend wider and frequently show a number of large protrusions accompanied with prominent F-actin on the Fc-chimera-protein-adsorbed test surfaces. **b** Prominent dot-like focal adhesions labeled with anti-vinculin antibody were accompanied by spots of F-actin in the cells on the EphA2-Fc-adsorbed surface. Doughnut-shaped focal adhesions accompanied with F-actin in their cores frequently appeared in cells on the ephrin-A1-Fc-adsorbed surface

Because HL60 cells from the VD-TNF group were significantly more adherent to the EphA2-Fc or ephrin-A1-Fc and Fc plus Matrigel-adsorbed surfaces than on the Fc plus Matrigel-adsorbed control surfaces, we examined whether ephrin-A and EphA activation induce morphological changes in focal adhesions and in actin filament organization in HL60 monocytes. Focal adhesions and F-actin were visualized by fluorescence labeling using an anti-vinculin antibody and fluorescence-tagged phalloidin, respectively. These labeling studies revealed dramatic cellular changes on both test surfaces compared to those on the control surface. Cells that extended more widely and frequently, possessed few large protrusions accompanied by prominent F-actin on the Fc-chimera protein-adsorbed test surfaces (Fig. 10a). Focal adhesions labeled by vinculin appeared indistinct in cells on the control surface. In contrast, a large number of clear and prominent focal adhesions, visualized as dot-like structures accompanied by clear spots of F-actin, appeared in the cells on the test surfaces (Fig. 10b). Moreover, doughnut-shaped focal adhesions with F-actin cores were frequently observed in cells on the Fc-chimera protein-adsorbed test surfaces (see also Additional file 3: Fig. S1): they appeared prominently in cells on the ephrin-A1-Fc-adsorbed surface compared to those on EphA2-Fc-adsorbed surface. These results indicated that EphA activation by ephrin-A1-Fc, and ephrin-A activation by EphA2-Fc, likely promote the formation of focal adhesions and F-actin reorganization in HL60 monocytes.

## Discussion

Eph receptors and their ligands, ephrins, primarily regulate cell adhesion and movement by modulating actin cytoskeleton organization mainly via Rho family GTPases [1]. Here, we demonstrated upregulation of EphA2, ephrin-A1, and ephrin-A2 during monocyte maturation in mouse bone marrow MNCs, and the induction of EphA2 and EphA4 as well as upregulation of ephrin-A4 during monocyte differentiation from HL60 cells. Thus, during monocyte differentiation/maturation, functional regulation by EphAs and ephrin-As is possibly acquired as a new feature, likely required for cell adhesion and movement. Several members of the EphA/ephrin-A subclass are known to be expressed in monocytes, macrophages, and monocyte/macrophage cell lines from humans and mice. Sakamoto et al. showed that human peripheral blood monocytes clearly express EphA1, EphA2, EphA4, ephrinA3, ephrinA4, and ephrin-A5 [34]. We have also shown that a human monocytic cell line, U937, expresses EphA1, EphA2, EphA4, ephrin-A1, ephrin-A3, and ephrin-A4; a mouse monocyte/macrophage cell line, J774.1, expresses EphA2, EphA4, ephrin-A1, and ephrin-A4 [20, 21]. These and our present findings indicate that the expression patterns of EphA/ephrin-A subclass members are certainly different between human and murine monocytes/monocyte-related cells, although the expression of EphA2, EphA4, and ephrin-A4 are common to both. It may not be important to note the difference in EphA/ephrinA expression patterns in monocytes and monocyte-related cells between mice and humans, as the cell surface markers that identify the monocyte subsets are largely different between these species [24]. Because EphA receptors promiscuously bind ephrin-A ligands within the same subclass [2, 8], the induction and upregulation of EphA/ephrin-A during monocyte differentiation and maturation should be highlighted.

We have previously demonstrated that EphA stimulation with ephrin-A1-Fc promotes cell adhesion through interaction with integrins and integrin ligands in the J774.1 monocyte/macrophage cell line and in the U937 monocytic cell line [20, 21]. In the present study, we have demonstrated that HL60 monocytes preferentially migrate and adhere to surfaces that contain ligands for EphA, ephrin-A, and integrins using a cell adhesion stripe assay with alternating stripes of ephrin-A1-Fc (to trigger EphA signaling) or EphA2-Fc (to trigger ephrin-A signaling) and integrin ligands with regions containing integrin ligands alone. HL60 cells formed stripes of different cell densities on ephrin-A1-Fc stripes as well as on EphA2-Fc stripes on the integrin ligand-coated surfaces. Naïve HL60 cells did not adhere to the Fc-chimera protein plus integrin ligand-coated surface, indicating that physical tethering by binding of EphA with ephrin-A1-Fc or by the binding of ephrin-A with EphA2-Fc, is not a likely driving force behind stripe formation because naïve HL60 cells expressed a certain member of the EphA/ephrin-A subclass at substantial levels. Thus, the findings of the cell adhesion stripe assay indicate that the arrest of cellular movement may be driven by EphA as well as ephrin-A signaling in interactions with integrins. To our knowledge, this is the first evidence of ephrin-A promoting cell adhesion to integrin ligands in monocytes. The crosstalk between Eph/ephrin and integrin/integrin ligands has been investigated to a limited extent in blood cells and their related cells. For example, EphA4 activation in human T-cells by ephrin-A1-Fc is shown to inhibit cell adhesion to fibronectin-, VCAM-1-, and ICAM-1-coated surfaces whereas ephrin-A activation by EphA2-Fc promotes cell adhesion to integrin ligand-coated surfaces [9]. Cell adhesion to fibronectin appears to be increased in dendritic cells derived from CD34^+^ positive progenitors following EphA2 activation with ephrin-A3-Fc, probably through a β1 integrin activation pathway [11]. In chronic lymphocytic leukemia cells, ephrin-A4 activation by EphA2-Fc significantly reduced cell adhesion to fibronectin-, collagen-, laminin-, ICAM-1-, and VCAM-1-coated surfaces [10]. EphA4 was previously shown to be physically associated with the αIIbβ3 integrin in resting platelets, and this association appears to support the stable accumulation of platelets on collagen surfaces even under flow [35]. Thus, we propose that EphA as well as ephrin-A signaling drives cellular movement arrest and promotes integrin-mediated cell adhesion, at least in monocytes and related cells. The present findings of the cell adhesion stripe assay also suggest that ephrin-A and EphA stimulation likely induces inside-out signaling to regulate integrin activation. This is because (1) additives such as chemokines that activate the signaling molecules mediating inside-out signaling by integrins were not used in this assay, thus, most integrins likely remain in their inactive form on the cell surface under this in vitro experimental condition, and (2) Rap1 GTPase, an essential molecule functioning as a molecular switch operating the inside-out signaling of integrins mediated by chemokine/chemokine receptor signaling [5–7], also operates in the Eph and ephrin signaling cascade [2, 3]. Further investigations will be required to determine Rap1 activation in EphA and ephrin-A signaling during monocyte adhesion to integrin ligands.

Furthermore, we have shown that EphA stimulation by ephrin-A1-Fc and ephrin-A4 stimulation by EphA2-Fc likely induce the formation and retraction of cell protrusions composed of F-actin in HL60 monocytes on integrin ligand coated surfaces. We have previously shown that by binding with ephrin-A1-Fc, the truncated EphA2 lacking almost the entire cytoplasmic region, promotes cell spreading and/or elongation along with promoting F-actin formation in U937 and J774.1 cells [20, 21]. It is well accepted that Eph and ephrin primarily regulate cell adhesion and movement by modulating the organization of the actin cytoskeleton mainly via the Rho family GTPases [1]. Thus, EphA and ephrin-A signaling likely regulate the formation of cell protrusions by modulating the activities of the Rho family GTPases in monocytes. We have also demonstrated that stimulation of both EphA and ephrin-A4 promotes the formation of prominent focal adhesions in HL60 monocytes on an integrin ligand coated surface. Moreover, doughnut-shaped focal adhesions that exhibit structural features similar to podosomes composed of F-actin dots surrounded by vinculin rings as visualized by phalloidin and vinculin staining [36], were frequently induced by EphA stimulation and occasionally by ephrin-A4 stimulation in HL60 monocytes on the integrin ligand-coated surface. Thus, molecular construction of focal adhesions is possibly different between those induced by EphA and ephrin-A signaling.

We have previously demonstrated that endogenous EphA stimulation with ephrin-A1-Fc promotes cell adhesion through interaction with integrins and integrin ligands and that, upon stimulation with ephrin-A1-Fc, the truncated EphA2 lacking almost the entire cytoplasmic region including the kinase domain, potentiates this adhesion and becomes associated with the integrin/integrin-ligand complex in J774.1 and U937 cells that express the truncated EphA2 construct [20, 21]. Here, we have demonstrated that ephrin-A1 stimulation likely promotes cell adhesion to the integrin ligand-coated surface in HL60 monocytes. Bone marrow stromal cells and vascular endothelial cells are known to express several members of EphA and ephrin-A subclasses [37–40]. Therefore, when monocytes encounter stromal and vascular endothelial cells, EphA as well as ephrin-A signaling is activated in monocytes, which possibly regulates integrin activities through several signaling pathways. Further studies are required to fully elucidate the EphA/integrin and the ephrin-A/integrin interactions underlying cell adhesion, migration, and infiltration of monocytes and their related cells.

## Conclusions

We have shown that EphA2, ephrin-A1, and ephrin-A2 expression was upregulated in murine bone marrow MNCs during monocyte maturation. In addition, EphA2 and EphA4 expression was induced, and ephrin-A4 expression was upregulated in HL60 cells along with monocyte differentiation. Using a cell attachment stripe assay, we have shown that stimulation of EphA as well as ephrin-A likely promoted adhesion to an integrin ligand-coated surface in HL60 monocytes. Moreover, EphA and ephrin-A stimulation likely promoted the formation of protrusions in HL60 monocytes. Notably, this study is the first analysis of EphA/ephrin-A expression during monocytic differentiation/maturation and of ephrin-A stimulation affecting monocyte adhesion to an integrin ligand-coated surface. Thus, we propose that monocyte adhesion via integrin activation and the formation of protrusions is likely promoted by stimulation of EphA as well as ephrin-A.

## Additional files

Additional file 3: Figure S1. Immunofluorescence micrographs showing vinculin and paxillin localization in HL60 cell from the VD-TNF group. HL60 cells from the VD-TNF group were cultured on a coverslip surface on which EphA2-Fc or ephrin-A1-Fc and Fc plus Matrigel were adsorbed. To visualize focal adhesions, cells fixed with 4% paraformaldehyde were incubated with 0.02% Triton X-100 in PBS and then with a mixture of an anti-human vinculin mouse monoclonal antibody (hVIN-1, Sigma-Aldrich) at a dilution of 1:200 and an anti-human paxillin rabbit monoclonal antibody (Y113, Abcam, Cambridge, UK) at a dilution of 1:250 in 1% BSA-PBS for 60 min at 32 °C. After washing with PBS, the cells were incubated with a mixture of Alexa 488-conjugated goat anti-mouse IgG (5 μg/mL; Molecular Probes) and Alexa 568-conjugated donkey anti-rabbit IgG (5 μg/mL; Molecular Probes) in 1% BSA-PBS for 30 min at 32°C. After mounting with PermaFluor (Thermo Fisher Scientific), fluorescence images of vinculin (green) and paxillin immunostaining (red) were photographed. (PPTX 976 kb)

## Abbreviations

BMCs: Bone marrow cells
BSA: Bovine serum albumin
F-actin: Filamentous actin
FBS: Fetal bovine serum
GAPDH: Glyceraldehyde-3-phosphate dehydrogenase
HBSS: Hank’s balanced salt solution
ICAM-1: Intercellular adhesion molecule 1
LFA-1: Lymphocyte function-associated antigen 1
Mac-1: Macrophage-1 antigen
MAdCAM-1: Mucosal vascular addressin cell adhesion molecule 1
M-CSF: Macrophage colony-stimulating factor
MNCs: Mononuclear cells
NSE: Nonspecific esterase
*p*: Probability
pen/strep: Penicillin and streptomycin
Rap1: Ras-related protein 1
RT-PCR: Reverse transcription polymerase chain reaction
SD: Standard deviation
TNF: Tumor necrosis factor
VCAM-1: Vascular cell adhesion molecule 1
VD: 1α, 25-dihydroxy-vitamin D_3_
VLA-4: Very late antigen-4
