# Supplementary figures and images for: EphA receptors and ephrin-A ligands are upregulated by monocytic differentiation/maturation and promote cell adhesion and protrusion formation in HL60 monocytes
Source: BMC Cell Biol. 2017 Aug 29;18:28. doi: 10.1186/s12860-017-0144-x (PMC5576293; doi:10.1186/s12860-017-0144-x)

## Slide 1
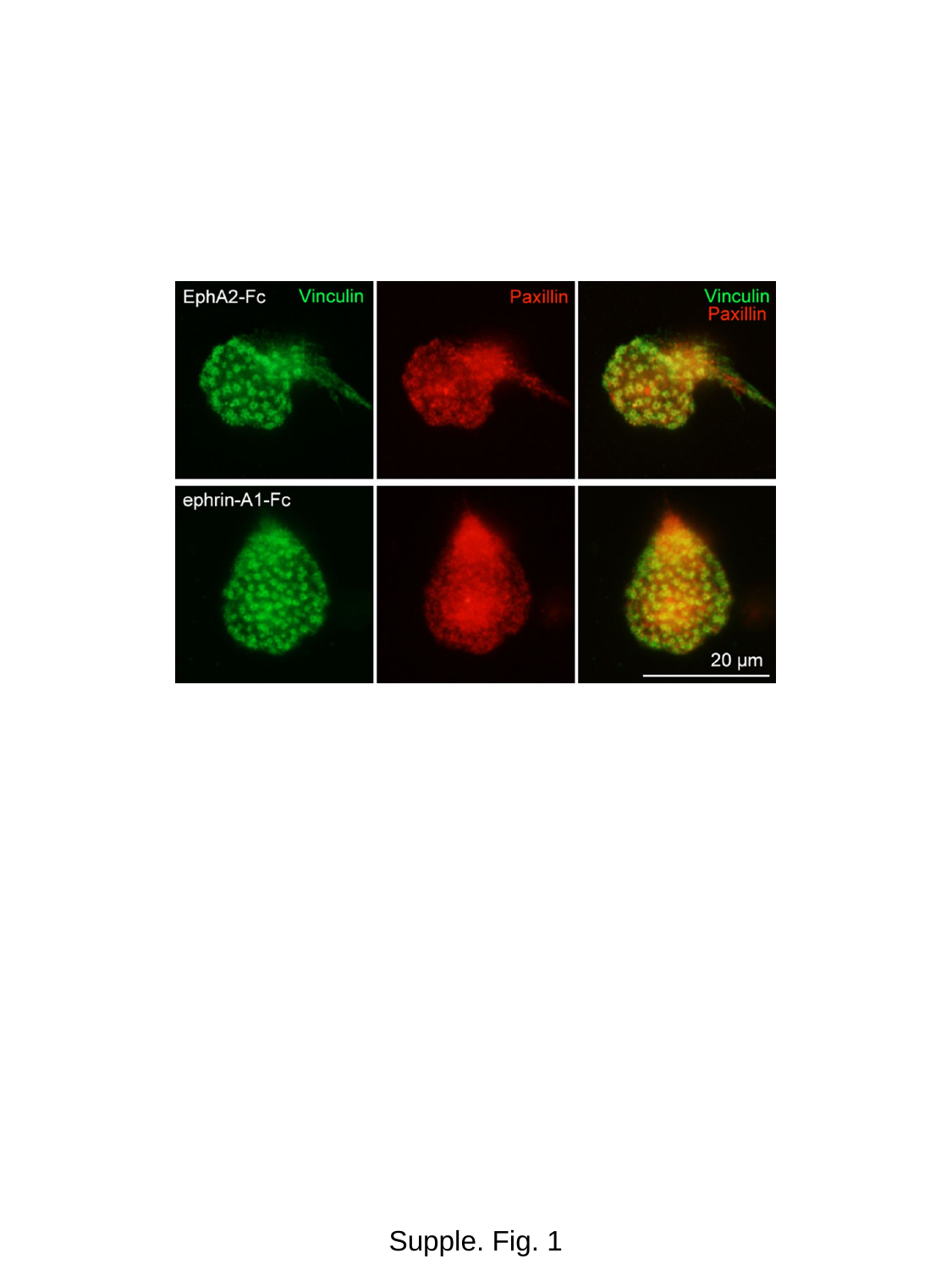

Supple. Fig. 1

Supplement: Supplementary file 3 — Immunofluorescence micrographs showing vinculin and paxillin localization in HL60 cell from the VD-TNF group. HL60 cells from the VD-TNF group were cultured on a coverslip surface on which EphA2-Fc or ephrin-A1-Fc and Fc plus Matrigel were adsorbed. To visualize focal adhesions, cells fixed with 4% paraformaldehyde were incubated with 0.02% Triton X-100 in PBS and then with a mixture of an anti-human vinculin mouse monoclonal antibody (hVIN-1, Sigma-Aldrich) at a dilution of 1:200 and an anti-human paxillin rabbit monoclonal antibody (Y113, Abcam, Cambridge, UK) at a dilution of 1:250 in 1% BSA-PBS for 60 min at 32 °C. After washing with PBS, the cells were incubated with a mixture of Alexa 488-conjugated goat anti-mouse IgG (5 μg/mL; Molecular Probes) and Alexa 568-conjugated donkey anti-rabbit IgG (5 μg/mL; Molecular Probes) in 1% BSA-PBS for 30 min at 32°C. After mounting with PermaFluor (Thermo Fisher Scientific), fluorescence images of vinculin (green) and paxillin immunostaining (red) were photographed. (PPTX 976 kb) [file 12860_2017_144_MOESM3_ESM.pptx]
